# Private land conservation through voluntary biodiversity conservation schemes: lessons from a payment for ecosystem services scheme in Finland

**DOI:** 10.1093/biosci/biaf155

**Published:** 2025-09-27

**Authors:** Khorloo Batpurev, Steve J Sinclair, Mar Cabeza, Kimmo Syrjänen, María Triviño, Heini Kujala

**Affiliations:** Conservation Biology Informatics Group, Finnish Natural History Museum, Helsinki, Finland; Department of Energy, Environment, Climate Action, Arthur Rylah Institute for Environmental Research, Heidelberg, Victoria, Australia; Global Change Conservation Lab, Faculty of Biological and Environmental Sciences, University of Helsinki, Helsinki, Finland; Department of Energy, Environment, Climate Action, Arthur Rylah Institute for Environmental Research, Heidelberg, Victoria, Australia; Global Change Conservation Lab, Faculty of Biological and Environmental Sciences, University of Helsinki, Helsinki, Finland; Finnish Environment Institute, Helsinki, Finland; Department of Biological and Environmental Science, School of Resource Wisdom, University of Jyväskylä, Jyväskylä, Finland; Conservation Biology Informatics Group, Finnish Natural History Museum, Helsinki, Finland

**Keywords:** PES scheme, forest, policy design, voluntary conservation, market-based instrument

## Abstract

In this study, we illustrate some of the most common challenges and pitfalls in payment for ecosystem services (PES) scheme design, through Finland's METSO program, a major forest-based PES policy in the European Union. We use four fundamental PES design concepts: conditionality, permanence, administrative targeting, and impact on social motivations. We find that METSO has primarily managed to avoid common design pitfalls. However, we identify some of the drawbacks of using timber volume as an ecosystem proxy for the conditions of payment. We then broaden our analysis with two issues that are novel in the literature: implications of achieving conservations targets such as the European Union Biodiversity Strategy 2030 through a private land conservation scheme such as METSO and implications of warming climate on the design of forest-based PES schemes. These issues call attention to the design of future policy instruments. Finally, we propose recommendations to policymakers on PES design.

The backbone of biodiversity conservation is the preservation of natural habitats within protected areas. Despite decades of efforts, only 16.64% of the world's land area has been protected (UNEP-WCMC and IUCN 2021). As biodiversity rapidly diminishes, conservation efforts outside protected areas are also needed. Private land conservation has become more relevant because of recent ambitious global and regional biodiversity commitments (EC 2020, CBD 2022) that cannot be met with strictly protected areas alone. For example, the European Union Biodiversity Strategy (EUBS) 2030 aims to protect 30% of Europe's land and sea areas by 2030 (EC 2020). Because much of Europe's land is privately owned and on small land parcels, the challenge is to devise effective policies with real influence over private land. To be widely adopted and effective, such policies will need to incorporate the needs and preferences of a large and diverse group of landowners and managers. Not only is this the case in Europe, but similar needs are arising globally (Abildtrup et al. 2021, Paletto et al. 2021).

## Challenges and pitfalls of PES schemes

Payments for ecosystem services (PES) are payments or improved market access to encourage private landowners to manage or reserve their land to benefit biodiversity or reduce the intensity of their impact on the environment. The number of PES schemes has increased rapidly in the last two decades (Song et al. 2023), despite reporting mixed results (Salzman et al. 2018). PES schemes are at the cusp of further acceleration as the corporate financial sector is starting to realize their business potential (Thompson 2021). PES is perhaps the pioneering precedent of many market-based instruments; trading in natural values, biodiversity credits and certification, carbon trading, and offsetting are examples of many market-based instruments that are emerging. As such, PES research and synthesis is important not only for the continued betterment of PES but also for the development and design of newly emerging market-based instruments.

Because of the complexity of social, economic, and ecological components of PES schemes, designing an effective scheme is a challenging task but a vital one, given that they are likely to play a major role in complementing the role of protected areas. The latest comprehensive account of PES schemes suggests that there are around 550 programs globally with a combined annual value of US$36–42 billion (Salzman et al. 2018). At the time, this was comparable to the global total spending on conservation, estimated at US$51–53 billion (CBD 2014). Despite the large diversion of funds into PES schemes, the effectiveness of these schemes remains unclear, and they are difficult to measure and compare with other biodiversity conservation tools because of the vast diversity of programs and a lack of documented details (IPBES 2019).

The PES literature is rich and manifold. The main program challenges that are repeatedly raised in the literature are summarized in columns 2–5 of supplemental table S1: The conditionality of PES in relation to paying for unreliable (or wrong) proxies for ecosystem services: The choice of proxy does not fully capture the desired ecosystem services or has perverse side effects (see the “Choice of ecosystem services proxy” section) such as misalignment with conditions of the payment. Motivation crowding: Adverse effects on people's motivation to act positively for nature conservation and to practice biodiversity-focused land-management practices (see the “Impact on social motivation” section). A lack of permanence and certainty: Most PES programs do not offer fixed-term or long-term contracts that landowners can rely on, resulting in ecosystem services provision that does not have a lasting legacy (see the “Permanence” section). And poor administrative targeting: Poor or unambitious protection targets caused by ulterior nonenvironmental motives (e.g., political or economic) rather than objectively seeking to harness better ecosystem services provision (see the “Governance” section).

The vast majority of literature reviews do not discuss program-level details that could reveal the application, successes, and failures of alternative PES designs because they analyze the PES literature rather than the schemes themselves. This gap inhibits improvements to the design of future schemes (Salzman et al. 2018).

## Structure and scope

In this article, we demonstrate how common challenges and pitfalls in PES schemes can be directly linked to on-the-ground program design. Insights that emerge from this discussion can provide a holistic view of the role and development needs of PES schemes in light of the global biodiversity policy ambitions. We do this by using the METSO program (for *Metsien Monimuotoisuus*, the Forest Biodiversity Programme for Southern Finland; box 1) in Finland, the European Union’s only forest-based PES with 17 years of history, as an example. In the “Common PES challenges illustrated through METSO” section, we summarize the strengths and weaknesses of the METSO program in relation to four common PES challenges described above and outline lessons learned from the program design. In that section, our aim is not to give an exhaustive overview of PES scheme challenges. Rather, we take well-defined and common issues to report on how they translate to on-the-ground program design features of METSO. On the basis of our review of the PES policy design literature overlap with our study (table S1), the four key challenges that we discuss do indeed appear to be ubiquitous, which makes our study broadly applicable to many PES programs.

Box 1. **METSO program summary: Basic introduction, mechanism and types of contracts.**

METSO, the Forest Biodiversity Action Program for Southern Finland, is a voluntary forest conservation program for nonindustrial forest owners in Southern Finland. Owners with valuable forests for biodiversity can volunteer their land to be protected for conservation and be compensated with a monetary sum. Starting in 2008, the aim of METSO is to protect 96,000 hectares (ha) of native forests by 2025. Access to the program is conditional on a minimum level of biodiversity value. The program is administrated by 15 regions across Southern Finland that independently assess forests against the biodiversity criteria. The compensation depends on the type of contract made.

Types of contracts: Private nature reserves involve permanent protection via a covenant applied to the land title, which remains in perpetuity. These covenants prohibit human intervention that reduces the biodiversity value of the forest. This excludes timber harvesting activities but allows recreational use of the land such as nature tourism and sports, mushroom and berry picking activities. Purchase by the state involves transfer of the land title to the state, in which case the monetary compensation includes the value of the land on top of timber value. Fixed-term nature reserves provide equivalent protections to private nature reserve but are enforced by a contract with a fixed term of 10 or 20 years.

Payment mechanism: The amount of compensation is calculated on the timber volume as a substitute for the economic loss of foregoing the income from selling the timber. For private nature reserves, the compensation is based on the economic value of the trees, using the average market prices (in euros per cubic meters) averaged across the past 3 years. For purchased by state, the compensation also includes the land value. For fixed-term contracts, the official compensation calculations account for aspects such as delayed revenue and reduced growth of forests due to not harvesting it at the end of the harvest cycle. The income from signing up to permanent protections and the 20-year fixed-term contracts is tax free.

**Figure 1.**
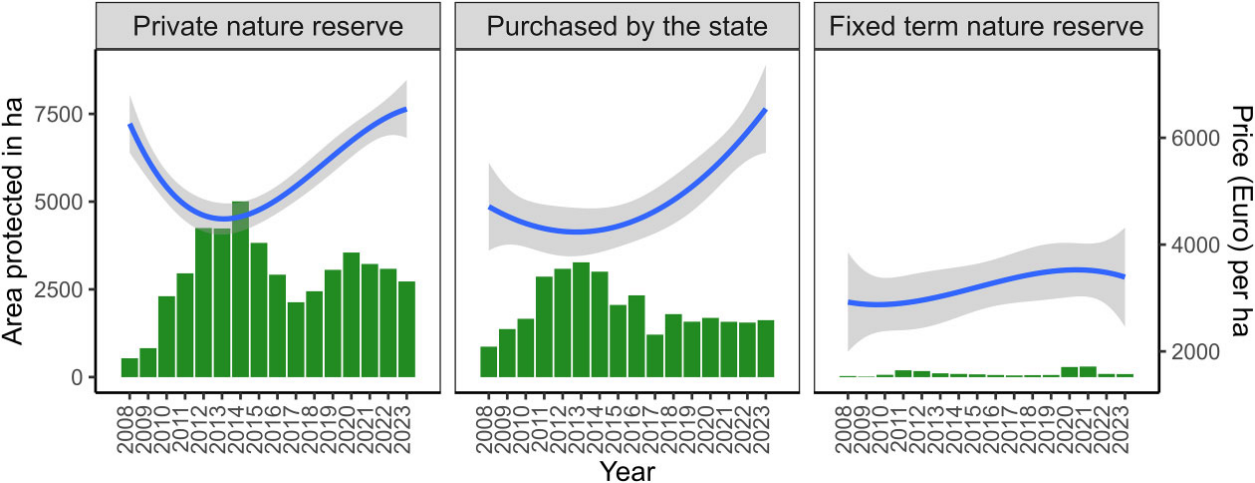
Summary statistics for the METSO program. The bars represent the area protected in hectares (the left **y**-axis), and the smooth function represents the cost of protection through time (euros per hectare on the right **y**-axis). The shaded area around the smooth function represents the standard error of the fitted linear splines model of the cost of protection per hectare.

Progress: Figure 1 shows the area of forest protected by these mechanisms through METSO since its commencement, up until 2023. To that date, METSO had spent approximately 400 million euros to protect a total of 80,313 ha (the sum of the bars in figure 1), toward a 96,000-ha target by 2025. The last decade has seen a consistent increase in the average cost of permanent protection, for both purchased by the state and private nature reserves, reflecting the increase in global timber value (the lines in figure 1) but also because of the directed focus on Southern Finland, where timber volume is generally higher per hectare.

Forests and owners: For most forest owners, the revenue from the forest does not constitute their main source of income. Most parcels protected under METSO are between 8 and 14 ha, which is smaller than the average size of forest holdings (30–48 ha; Kulju et al. 2022). This indicates that parcels protected are likely only a proportion of average forest holdings (between 16% and 47%).

Below, we further introduce two less commonly discussed topics in the PES design literature (table S1), which are topical in the rapidly developing policy environment. In the “Practicalities of using PES schemes to meet ambitious conservation policy targets” section, we evaluate the cost implications of using METSO-like tools to achieve ambitious biodiversity policy goals, focusing on the EUBS 2030 targets. Finally, we discuss the emerging challenges that externalities such as climate change pose for voluntary compensation schemes, which, so far, has been an absent topic in the PES literature (see the “Externalities in PES” section). We summarize METSO-related details in box 1 but discuss specific design features throughout the following sections under respective themes.

Although we examine the effectiveness of all types of PES, the METSO example narrows the focus to direct land protection, excluding biodiversity offsets and non-land-tethered values such as water and air quality. METSO nevertheless serves as a useful and timely case study, because the program has an extensively documented history, and as a voluntary private land conservation program, it represents one of the key mechanisms to achieving the EUBS 2030 targets. We focus on four common PES design-related challenges; however, there are several others that are not addressed in this article (i.e., are outside the scope), two of which are limited or no additionality, a lack of extra benefits in addition to the existing actions and behaviors of landowners that increase the provision of ecosystem services or biodiversity status despite the PES funding (Persson and Alpizar 2013; also column 5 of table S1), and a leakage effect, the risk that a PES scheme targeting one location manages to increase ecosystem services provision at local scale, but this, in turn, leads to exacerbation of land-use pressures elsewhere in the world (column 5 of table S1).

## Approach and rationale

Our analysis was based on a combination of a literature review of PES schemes to identify common challenges faced by programs, followed by a close examination of METSO through data focused on the program. We followed the design criteria given in some of the seminal papers on PES schemes (Engel et al. 2008, Wunder et al. 2008, Pagiola 2011, Wunder et al. 2020) and considered their overlap with and relevance to the METSO program. We further confirmed the prevalence of these challenges in the program-based PES literature (table S1), compiled using search terms *PES scheme effectiveness for biodiversity conservation, Payments for conservation, Payments for environmental services, Payments for ecosystem services* in combination with the terms *design* and *effectiveness*. We used full text search on publications after 2000 using Google Scholar (English language) without any discipline specifications on two separate occasions, the last time on 14 May 2025. A small number of additional references were identified through a first-order snowball process from the articles that were identified through this search. The rest of our analysis is based on a combination of data sources including publicly available data on the METSO program, published data on timber volume in forest stands (Virkkala et al. 2022), published data on the conservation priority of forests produced to identify areas important for forest biodiversity conservation in Finland (Forsius et al. 2023, Mikkonen et al. 2023), and spatial data on public lands in Finland (supplemental table S2). We carried out area calculations for the EUBS targets using GIS (geographical information system) software ArcMap 10.8 (ESRI 2020).

## Common PES challenges illustrated through METSO

In this section, we illustrate the four challenges that many PES schemes face (described above), using Finland's METSO program as an example: the choice of ecosystem services proxy, otherwise known as *conditionality*; the impact on social motivation; permanence; and governance.

### The choice of ecosystem services proxy: Compensation based on the loss of economic opportunity rather than on a biodiversity outcome

Most PES payments are calculated on the basis of one of two quantities: either by input, where payments are based on people's efforts or management actions, or by outcome, where people are compensated for the actual biodiversity outcome that results from their efforts (Engel et al. 2008). The return on conservation investments is higher for outcome-based PES than for input-based PES (Kroeger 2013, McDonald et al. 2018, Wuepper and Huber 2022). However, outcomes are more difficult to measure, and comprehensive accounting of them comes with high costs. Therefore, many PES schemes resort to using raw natural resources as the main unit of compensation or payment to reduce administrative costs (Brownson et al. 2020, Wuepper and Huber 2022).

**Figure 2.**
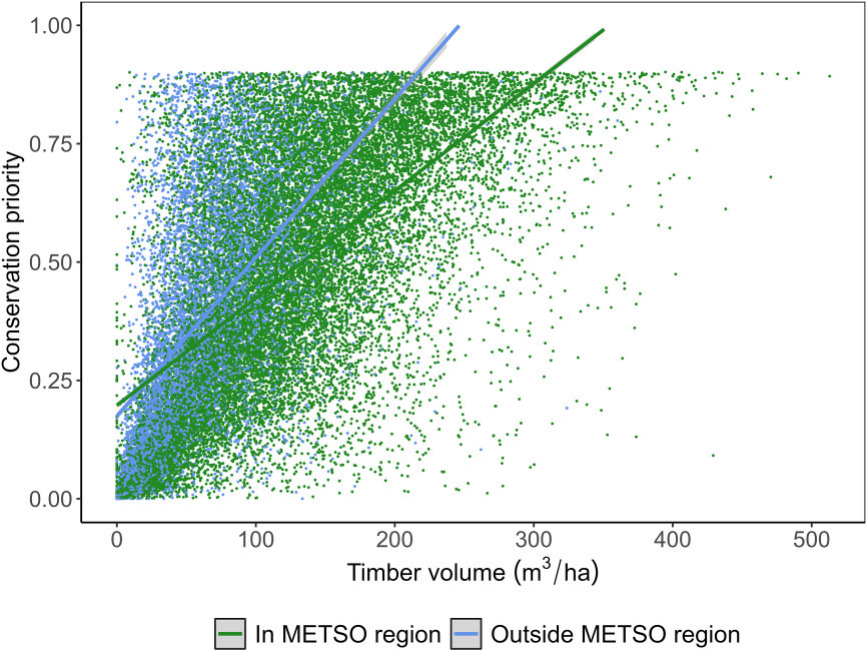
Correlation between timber volume (in cubic meters per hectare) and conservation priority inside and outside the METSO region. Conservation priority is given on a scale from 0 (lowest priority) to 1 (highest priority) on the **y**-axis. Each dot represents 1 of 30,000 randomly selected, approximately 1-hectare unprotected forested sites. Source: The data on volume are from Virkkala and colleagues (2022), and the data on priority are from Forsius and colleagues (2023).

The current METSO compensation is based on timber volume. Old-growth forests have high timber volume, and therefore, offer high biodiversity values. Figure 2 shows that timber volume and conservation priority of Finnish forests are indeed correlated. Timber volume is naturally higher in southern and central Finland (in the METSO region) than in northern Finland (outside the METSO region); however, the correlation between conservation priority and timber volume is very weak at high conservation priority sites (closer to 1 on the *y*-axis), particularly in the METSO region. This is partly because some high conservation priority values relate to ecosystems that are naturally low in tree density. This means that landowners with sites that are naturally low in timber volume but high in biodiversity value are not proportionally rewarded for their contribution to biodiversity conservation, because their compensation is only related to the volume of wood.

There are many aspects of biodiversity that are not adequately accounted for by timber volume. This includes the amount of deadwood, understory structure, species diversity, and landscape features such as patch size and connectivity to other forest sites. Even though these features are part of the site selection criteria in METSO, a forest owner is not financially compensated for fostering and protecting these features. Deadwood is a particularly important biodiversity asset, because it provides habitat for many threatened forest species, including fungi, insects, and birds (Ranius et al. 2016, Parisi et al. 2018, Lofroth et al. 2023); therefore, it is a good indicator of forest biodiversity (Lassauce et al. 2011). Approximately 25% of all boreal forest species live in deadwood, and the volumes are higher in forests that are not intensively managed (Stokland et al. 2004). Site connectivity potential is another important feature that could be incorporated into the payment scheme, especially when demand for PES contracts exceed the uptake (discussed in the “Recommendations” section). Making the compensation proportional to site connectivity also incentivizes neighboring properties to conjoin conservation efforts (Reeson et al. 2011).

The missing direct link between PES and biodiversity values is one of the main reasons it is difficult to assess the effectiveness of PES schemes. Our literature review has revealed that there are only a few PES schemes that incorporate biodiversity values into the way compensation is calculated. This is despite there being potentially innovative approaches available, such as reverse auctions (Naidoo and Adamowitz 2005, Murdoch et al. 2007, Ens 2012) and methods for assessing aggregated biodiversity values of different kinds (e.g., Sinclair et al. 2021).

### Impact on social motivation

PES schemes introduce many ethical and cultural dilemmas at community and society levels. Gomez-Baggethun and colleagues (2010) suggested that the cultural impacts of PES may include long-term changes in a landowner's quality of life, independence, attitudes or belief systems, and security, along with social changes such as the empowerment of women, changes to community identity, and changes in behavior and motivations for conserving nature. These changes introduce numerous ethical dimensions to the decision to implement PES schemes. One major ethical concern centers on the anthropocentric and utilitarian framing of most PES schemes. Many schemes assume that nature is there to service humans (i.e., ecosystem services assumption). The commodification of natural environments may force a utilitarian ideal on environmental protection, which could be creating a new social norm or expectation that protecting the environment should result in or require monetary reward. This is known as the *motivation crowding out effect* in literature (Rode et al. 2015).

On the other hand, the increasing globalization of the natural resources market means that landowners or producers in some parts of the world could miss out on financial opportunities by being forced to rely on said relational values toward nature protection while landowners on the other side of the world get paid to reduce the impact of the same product on biodiversity (Luck et al. 2012). Therefore, the balance between fostering relational values in landowners and economic levers to achieve biodiversity conservation on private land must be struck carefully.

The best-case scenario for a PES scheme is to protect the environment from degradation either in perpetuity (via permanent mechanisms) or to delay land-use pressures while promoting a more nature-oriented attitude by financially supporting landowners and managers. In the worst-case scenario, as soon as financial incentives stop, unsustainable land use returns, and the PES scheme achieves no long-term positive change (Engel et al. 2008, Wunder et al. 2008, Erbaugh 2022). Not all PES schemes result in these undesirable outcomes, and there have been many recent reports of improved nature-oriented intrinsic values (motivational crowding in), even after payments have stopped for environmental services (Jayachandran et al. 2017, Lliso et al. 2022, Vorlaufer et al. 2022, Blanco et al. 2023).

Thus far, only one study has investigated whether there have been any motivational crowding effects of METSO (Primmer et al. 2014). This research showed that, by adopting a hands-off voluntary approach and leaving the decision-making to the forest owners, the program did not reduce the landholders’ motivation toward conserving nature. Another study looking at how forest owners’ value systems affect their motivation to protect forest biodiversity showed that, in Finland, forest owners with stronger motivation to protect nature already act in ways that are additional to signing up to an incentives scheme (Koskela and Karppinen 2021), supporting the claim that there is little danger of motivation crowding out risk in the METSO program. However, it is important to acknowledge that data collection in this work was based on a random sample and was not targeted at METSO-contracted forest owners.

One of the major achievements of the METSO program so far has been the process of repairing the frayed relationship between nonindustrial private forest owners and the state, after the top-down approach adopted by the Finnish government as Finland became a member of the European Union in 1995 and joined the European Union’s Natura 2000 program. Under Natura 2000, private land was acquired for conservation with little or no negotiation with landowners, causing substantial conflict and distrust between the landowners and the authorities (Kati et al. 2015, Blicharska et al. 2016). In many ways, METSO is still dealing with the social ramifications of the Natura 2000 program.

### Permanence: Contract arrangements

The details of contractual arrangements affect the success of PES schemes significantly (Pagiola 2011, Mahanty et al. 2013, Wunder et al. 2020), especially when it comes to the tenure, duration, and conditionality of the contracts. A major obstacle to the efficiency of PES schemes stems from the variable and often short length of contracts, because of uncertainty associated with funding, and the lack of temporal legacy of improved ecosystem services provision. The insecurity of these types of contractual arrangements often hinders the commitment of local people, therefore reducing the adoptability and success of PES schemes (Erbaugh 2022). Seven case studies from Mexico, Brazil, Uganda, Mozambique, the Phillipines, Indonesia and Nicaragua, and Colombia under the REDD+ program indicated that uncertain contractual terms hindered the effectiveness and sustainability of ecosystem services provision (Mahanty et al. 2013).

Because of the voluntary nature of most PES schemes and because they are often based on the landowner's efforts, most PES contracts globally are temporary (Mahanty et al. 2013). METSO is an exception, in the sense that 99% (figure 1) of the funding used by the environmental authorities (the Finnish Centers for Economic Development, Transport, and the Environment) for the implementation of the METSO program has been spent on permanent protection and less than 1% on temporary contracts. By focusing on permanent contracts and making fixed-term contract durations known to the forest owners prior to their signing up, METSO is largely safe from the pitfalls experienced by other PES schemes.

Fixed-term temporary contracts can be a useful governance tool, especially if they are used as a stepping stone to secure permanent protection through increased landowner motivation at the end of the contract (Sironen et al. 2020) while allowing time for negotiation for the owners to transition to a permanent contract. This is likely to be especially important in Finland, given the complex multigenerational family ownership of many forest parcels. Up until 2022, there were 206 temporary contracts signed under METSO, covering 1641 hectares (ha) of forests. Hänninen and colleagues (2021) found that 54% of environmental forestry subsidy agreements signed under the METSO program are renewed, a relatively high rate that does not suffer from the major issues of motivational crowding out described above.

### Governance: Administrative targeting

A considerable proportion of threatened ecosystems globally are now located in and around densely populated areas and overlap with high land values and high pressures on natural resources (Sierra and Russman 2006). This nonrandom distribution of biodiversity assets and land-use pressures creates a myriad of administrative problems for PES schemes such as METSO. Primarily, it creates a spatial bias in conservation allocation because low-pressure areas tend to be protected disproportionately (Wunder et al. 2020), especially when PES policy is dominated by political and economic motives (Rosa da Conceição et al. 2015).

**Figure 3.**
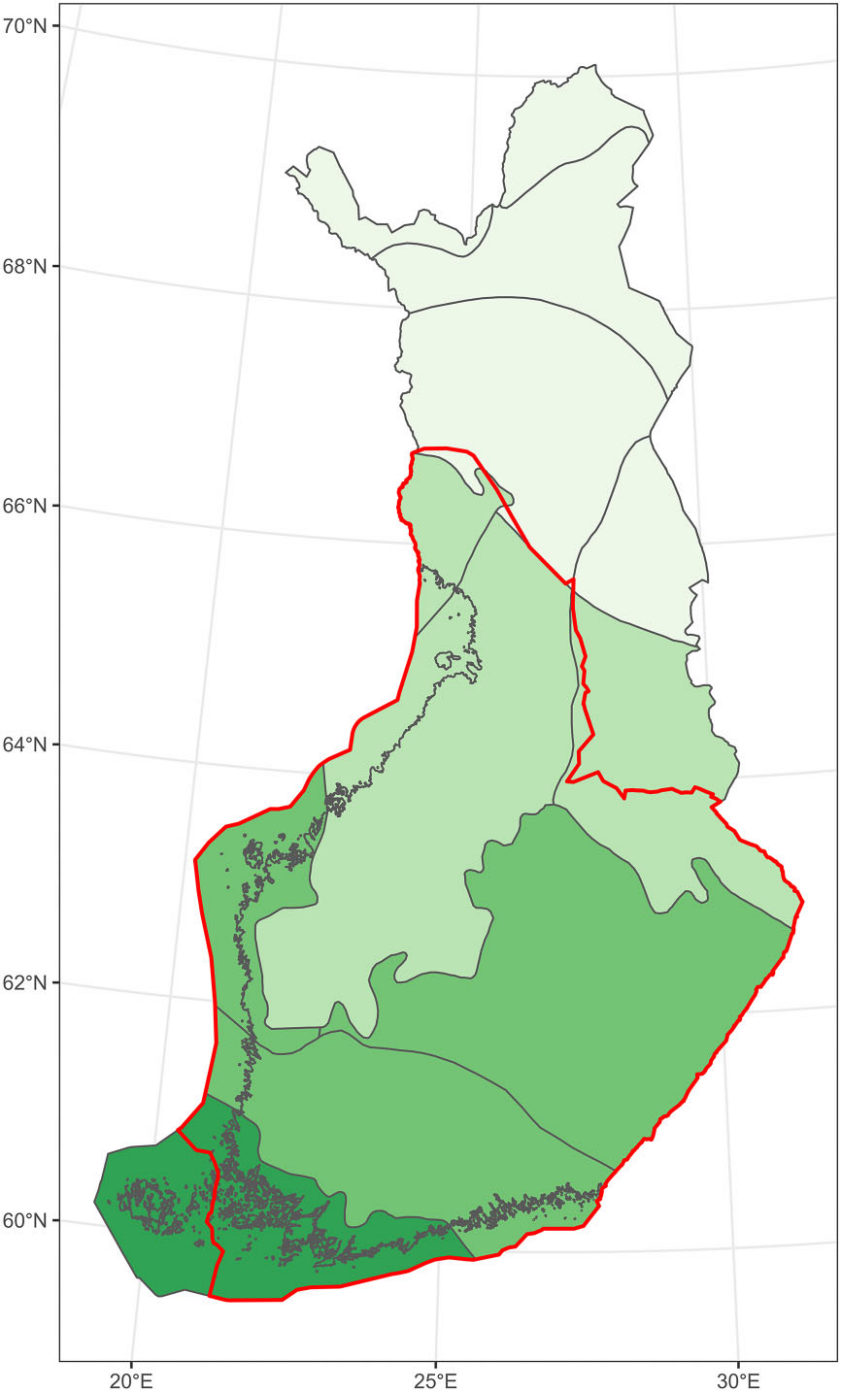
The inner line delineates Southern Finland, which is the operating area for METSO program. Shades indicate the gradient in boreal biogeographical zones: hemi boreal (darkest shade), southern boreal, middle boreal, northern boreal (lightest shade).

METSO largely avoids this trap through two key design features. First, the program is focused on southern and central Finland (figure 3), where the majority of high-pressure areas from timber harvesting and urbanization overlap with high conservation priority areas. Had METSO been focused on low-hanging fruit, the majority of the contracts would have been focused on northern Finland, a low-pressure and low merchantable timber-producing area where most of Finland's protected areas are located. Second, the administration of the program through regional Centers for Economic Development, Transport, and the Environment guarantees that contracts are evenly distributed across southern and central Finland, further reducing spatial biases.

However, the overlap between conservation priority and high-pressure areas also creates difficulties in the administration of METSO (figure 3). This is due to the highly fragmented nature of the METSO region (Kouki et al. 2010), where forest holdings are smaller in size than those in northern Finland. Because forest protection contracts are negotiated on an individual basis, the administrative cost per hectare is higher in high-pressure areas. Another difficulty related to program administration is that because METSO is administered by multiple regions with different funding allocations, there is often a mismatch in conservation demand and uptake. Currently, in many areas, forest owners with high-value forests are unable to secure a protection contract because of limited funding in their region.

Governance structure and administrative capacity features determine the path of success or failure of PES (Ferraro 2017, Wunder et al. 2020), especially in the context of ambitious global biodiversity targets. We discuss what the practical implications may be for METSO in the context of EUBS 2030 in detail in the “Practicalities of using PES schemes to meet ambitious conservation policy targets” section. In our view, the above three features of the METSO program track well against the common challenges that PES schemes face elsewhere. Therefore, it is important that these continue to be maintained in the program, if METSO is to play a role in meeting EUBS target.

## The practicalities of using PES schemes to meet ambitious conservation policy targets

The role of PES schemes in the global efforts to halt biodiversity loss remains debated (Salzman et al. 2018). The importance of voluntary conservation on privately owned lands is well established, but to what degree can such schemes deliver biodiversity outcomes? We explore the cost implications of using PES-like schemes to meet ambitious biodiversity targets such as the EUBS 2030.

The EUBS’s 30% protection target applies to all biogeographical regions, including the boreal region, which is mostly located in Finland and Sweden. There are no official country quotas, but following the equal-burden-sharing (Armstrong 2019) principle, all countries in the boreal region are likely expected to protect 30% of their boreal habitats. Furthermore, one-third of the 30% target (i.e., 10% of the total area) is to be strictly protected, a target most likely be inclusive of current protected areas (referred to as *the 10/30 target*). These directives are currently nonbinding, which means member states are not legally obligated to implement them. These targets pose challenges for Finland, because most of the high biodiversity areas are in southern Finland, where 71% of the forested land is privately owned (Hanski 2000, Kallio et al. 2008, Mikkonen et al. 2023). Therefore, if EUBS 10/30 is to be achieved, it will likely have to be through PES schemes such as METSO or land acquisition (Winkel et al. 2022).

In table 1, we explore the practical implications of reaching the EUBS 10/30 target using METSO under two conservation scenarios (the columns in table 1): where the 10/30 target is met nationally and where it is met at a regional scale. We estimated the remaining areas needed to achieve the 10/30 target on public and private land in Finland. For this, we used a previously developed conservation plan (Forsius et al. 2023) to determine the top 10% and 30% priority areas of the boreal bioregion in Finland under the assumption that Finland will strive to protect these high conservation value areas first. To factor in already strictly protected areas, we used the results of Kuusela and colleagues (2022). They calculated that 9.8% of all forested areas, including both high- and low-productivity forests, are protected in Finland, the majority of which are in northern Finland. Finland is currently in the process of deciding which types of areas will count toward other effective conservation measures. Because there is currently no comprehensive national dataset of other effective conservation measures, we opted to include only strictly protected forested areas in our analysis, whereas areas of potential other effective conservation measures such as biodiversity conservation sites on forestry land that are already in place were excluded. Although this gives only a partial picture of the current protection status, our analysis allows us to illustrate the scale of additional conservation measures needed to meet the EUBS targets and how this is split between private and public lands.

**Table 1.** Proportion of land that needs to be protected in addition to the already-protected areas in private tenure versus public land, in percentage and hectares, rounded to the nearest thousand hectares.

	National priorities				Regional priorities			
	10% strict protection		30%		10% strict protection		30%	
Sector	Percentage	Area (in hectares)	Percentage	Area (in hectares)	Percentage	Area (in hectares)	Percentage	Area (in hectares)
Private	83	36,000	82	4,627,000	90	1,034,000	83	4,730,000
Public	17	7600	18	1,036,000	10	115,000	17	940,000

*Note: National priority* refers to the 10% and 30% protection targets being met nationally, and *regional priority* is what is being met for each of the administrative regions in Finland.

Our analysis suggests that, to meet the 30% overall protection target nationally, the Finnish government would need to acquire 4.6 million ha of privately owned forests (including the 10% strictly protected area quota). To meet the EUBS 30% target for boreal forests shared equally by the regions, Finland will likely have to protect 4.7 million ha of privately owned forests (including the 1 million ha under the strictly protected quota). These figures are in addition to the already protected areas. Our analysis complements that of the Finnish Nature Panel (Kotiaho et al. 2021), who also advised that the 10/30 target should be met in all administrative regions of Finland to ensure a better representation of all forest types, including, for example, the more southern herb-rich forests and broadleaf deciduous forests.

These figures illustrate the massive scale of private land protection needed to achieve the EUBS targets in Finland: Between 80% and 90% of the areas that need to be protected fall on privately owned land if this kind of prioritization (Forsius et al. 2023) is used. The area that is privately owned under the 10% strict protection target varies significantly when calculated at a national or regional level (nearly a thirtyfold difference), whereas, with the 30% target, the area that needs to be protected that falls on privately owned land is nearly the same (approximately 4.6–4.7 million ha) at either the national or the regional level.

To reach the 10% strict protection target, it is likely that the forested areas on privately owned land (36,000 ha) will have to be secured via a mechanism such as METSO. Using the cost of the last 5 years’ METSO compensation rates for permanent protection contracts (6314 euros per ha) and the area estimates required in hectares (table 1), the future budget of voluntary conservation programs is estimated to be around 225 million euros to reach the 10% strict protection target nationally, and 6 billion euros to reach the target regionally. This estimate assumes that future programs will continue to use timber volume as the basis of compensation and that timber growth and price remain constant, making these estimates the bare minimum. 36,000 ha of forest protection is nearly one-third of the current protection target for METSO over the 17-year period. It is unlikely that timber prices will remain the same in the near future; since the Ukrainian war and the trade sanctions on timber exports from Russia, Finland's domestic timber demands have increased dramatically (Dzian et al. 2022). Internationally, global timber prices could increase by two to four times by 2040 relative to 2020 prices (Daigneault et al. 2022).

To protect the remaining 20% of the boreal bioregion, Finland is likely to choose a less stringent and a less costly mechanism than METSO, such as other effective conservation measures in the form of forest subsidies. On the basis of the mean of the last 5 years of forest subsidization rates for protection of forest from timber harvesting (2448 euros per ha) under the Metka program (the Act on a Temporary Forestry Incentive Scheme; Koskela et al. 2024), and the area estimates required in hectares (table 1), the future budget to reach 30% overall protection target may range between 13 billion and 15 billion euros at a national and regional level respectively.

## Externalities in PES: How climate change may induce currently unforeseen challenges

Climate change is a major external factor that affects PES scheme successes or efficiencies in all corners of the world. It introduces a multitude of challenges. In the present section, we focus on the biophysical implications of climate-induced changes in boreal forests and how these could affect the future protection targets for schemes such as METSO.

The implications of climate change effects in the boreal bioregion may manifest in divergent and nonlinear ways. On one hand, the increasing temperatures could increase tree growth in the near future (Alrahahleh et al. 2016, Pukkala et al. 2021, Triviño et al. 2023a), particularly in the northern boreal region (Triviño et al. 2023b). On the other hand, the increasing frequency and intensity of extreme events such as windstorms, wildfires, and insect and pathogen outbreaks could increase tree mortality (Venäläinen et al. 2020, Pukkala et al. 2021) in the long term. The interaction of these possible events into the future only adds to the complexity of predicting the net effect of climate change on ecosystem services provision and, therefore, PES schemes. Here we focus on a single factor, the effect of an increase or decrease in timber volume growth on future METSO compensation because of climate induced changes.

**Figure 4.**
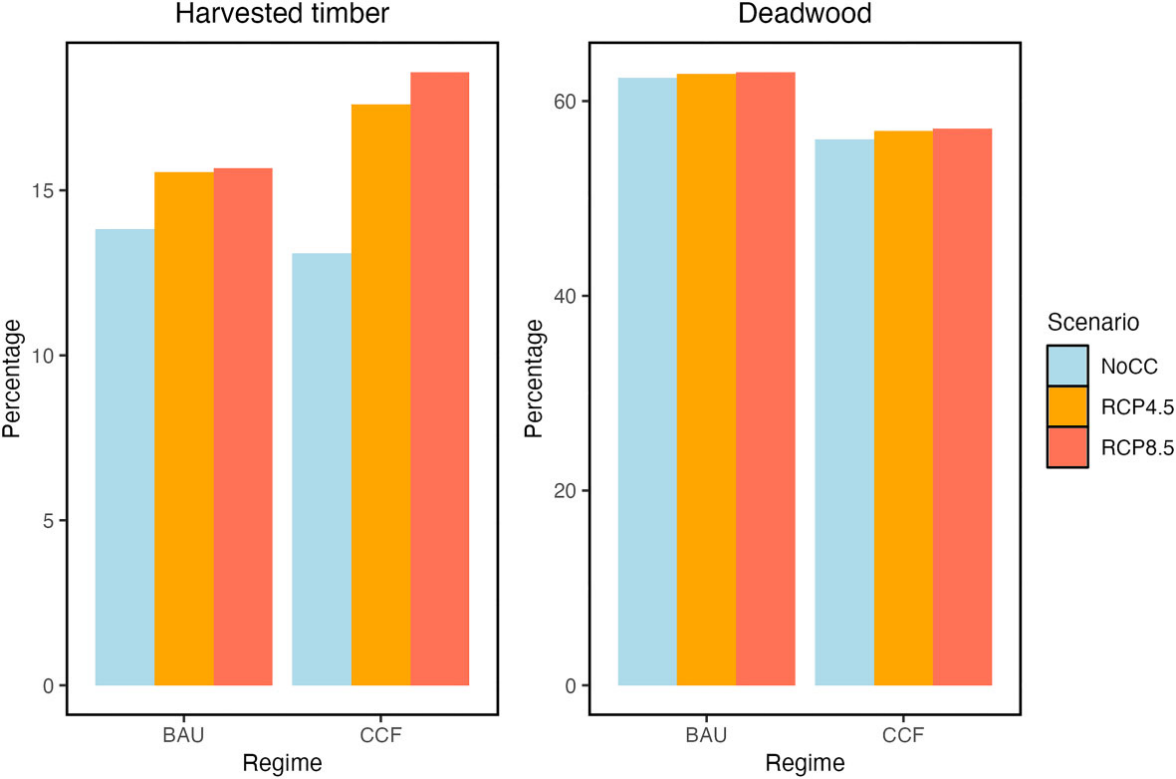
Predicted amount of harvested timber and deadwood over 2021–2041 period under business as usual and continuous cover forestry harvesting scenarios under three climate change scenarios (no climate change, RCP4.5, and RCP8.5). Source: Adapted results from Triviño and colleagues (2023a).

We use a recent publication by Triviño and colleagues (2023a), where they estimated that there would be an approximately 2% increase in harvested timber under RCP4.5 and RCP8.5 (the Intergovernmental Panel on Climate Change's Representative Concentration Pathways) under a business-as-usual management scenario (i.e., even-aged forestry with final clear cut) because of an overall increase in timber growth in this period relative to a no-climate-change scenario in the next 20 years (figure 4) in Finland. Taking the mean timber stocking volume per hectare estimates in southern Finland (141 cubic meters per ha) from Korhonen and colleagues (2021) and the mean METSO compensation (6314 Euro per ha) estimates, if we were to translate the 2% increase in timber volume into monetary terms, it would mean a difference of around 6.8 million euros extra that would be required to protect 36,000 ha to achieve the 10% strict protection quota for EUBS. Alrahahleh and colleagues (2016) predicted even a higher timber volume growth, with increase of 10% and 12% in southern Finland over 2010–2039 period under RCP4.5 and 8.5, respectively.

At the same time, the same studies predict that in the mid- to long term (70–100 years), tree mortality could increase because of drought stress and other extreme weather events and insect outbreaks (particularly under RCP8.5) that could decrease timber volume. In this case, the cost of compensation based on timber volume could be less, contrary to the above estimate. They also predict a slight increase in deadwood under both RCP4.5 and 8.5 (figure 4; Alrahahleh et al. 2016, Venäläinen et al. 2020, Pukkala et al. 2021, Triviño et al. 2023a), which technically means increased biodiversity value, given that deadwood is a critical resource for many forest species (Stokland et al. 2012).

Overall, the combination of these events imposed by climate change impels a highly uncertain future for voluntary forest compensation schemes such as METSO. Climate change implications for future design and implementation of these types of schemes are, so far, an absent topic in the PES literature.

## PES schemes moving forward

The key question around PES schemes is whether they are the most cost-efficient way for future conservation. Many researchers argue that schemes are not always the most cost-efficient mechanism for conservation as success relies on landowners’ willingness to sell (Knight et al. 2011). On the other hand, voluntary conservation agreements have been found to contain many very important components for the successful implementation of biodiversity conservation (Horne 2006, Miljand et al. 2021). The traditional top-down approach to achieving global biodiversity targets may be easier administratively and cost less fiscally than a voluntary approach using PES. However, the lessons from past top-down approaches (e.g., Natura 2000) demonstrate that land expropriation is likely to exact a heavy price in the form of negative public attitudes to conservation and loss of trust in institutions (Hiedanpää 2002). Nevertheless, the voluntary strategy in the form of PES is clearly not a straightforward approach, as is shown in this study.

The second prominent question about costs and benefits is around what PES expenditure could buy elsewhere, such as in the global south, where some countries face crippling poverty and where the consumption of natural resources is a matter of survival and livelihoods of people on the margins. Many developed countries choose to offset their impact on biodiversity outside their borders where gains are cheaper to secure. An example is neighboring Norway donating US$3.68 billion to the REDD+ program in 2020, targeting developing countries in tropical regions (Westholm et al. 2011, Morita and Matsumoto 2023). The Finnish case is, however, unique in that the boreal bioregion is a critical component of the continent's biodiversity representativeness, and therefore, offsetting externally would not be a viable option without trading away one asset for another.

### The role of PES schemes in Europe

Programs such as the METSO are likely to be an integral part of achieving global biodiversity conservation targets as protected areas alone are not sufficient for meeting these, let alone halting the biodiversity crisis. METSO has been important in shifting the public sentiment about forest conservation in Finland. The design was strongly motivated by the trauma caused by the top-down land acquisition approach in building the Natura 2000 network (Hiedanpää 2002), which created resistance toward conservation among landowners in Finland and more widely in Europe (Tiebel et al. 2021). By allowing landowners to voluntarily offer their forests for protection, the program attempts to reverse the power dynamics traditionally seen in conservation. The trust relationship between stakeholders and the government is often underrated, but it plays a vital role in successful PES schemes (Matthews and Missingham 2009, Ford et al. 2012).

Given the METSO program's history and continuity, cumulative corporate knowledge is likely to be essential to achieve ambitious targets such as the EUBS 2030. The present study illustrates that there are some noteworthy practical challenges for PES schemes that are likely ubiquitous. Choosing the right ecosystem services proxy is crucial; a compensation mechanism based on a market-related commodity rather than biodiversity outcome leaves PES schemes exposed to pitfalls such as price inflation of conservation contracts without the proportional increase in conservation outcome. The added complication of externalities such as climate-induced effects on the future cost of conservation contracts and to the ability or willingness of landowners to participate need to be incorporated into future PES planning and biodiversity targets, particularly in the global south and in agricultural PES schemes. If biodiversity conservation targets such as the EUBS 2030 are to be achieved, PES schemes such as METSO must be expanded massively (10–50 times), especially in the case of high private ownership and the highly parcellated nature of land tenure in Europe.

There are some good news stories that should be shared from METSO—namely, the legacy of the scheme that will leave over 90,000 ha of forests permanently protected, the careful strategy that aims not to tamper with landowners’ social motivation around nature conservation, and the good administrative targeting of high conservation priority areas.

Climate change implications for the timber volume-based compensation mechanism proves a challenging reality for Finland's future conservation efforts if METSO was to be part of their commitment to EUBS 2030 target. This challenge is likely to be a reality for many other countries in Europe.

Biodiversity loss and ecosystem degradation issues cannot be solved by PES schemes alone, so these should be implemented alongside other policy measures in a complimentary manner. For example, one of the main avenues for climate change mitigation is the emerging carbon market, where retaining mature forests from timber production will play an important role and forest owners may have the option to earn income from keeping their forest plots intact. How the carbon market fits into forest conservation, whether there might be synergies that could optimize both biodiversity and climate benefits, and whether they may have opposing incentives targeting landowners are topics under discussion (Kangas and Ollikainen 2022). Another development in this space is the expansion of wind farms in forested landscapes in Europe, which may create a competing demand for forest conservation (Balotari-Chiebao et al. 2023).

Despite the unified aim to conserve nature, it is widely documented that environmental policies create competing opportunities and demands for landowners (Lister 2011, Gulbrandsen 2014, Lambin et al. 2014). In the case of Finland, there are some clear synergies with other environmental policies. An example is forest subsidies granted by the Ministry of Agriculture and Forestry in Finland (the Metka program). These reward forest owners who elect to keep parts of their forests intact from forestry activities and leave waterways undisturbed. This subsidy policy is not mutually exclusive with METSO sites, and the two can work in tandem. Broadly speaking, environmental subsidies often have mixed effects on biodiversity conservation; this topic is large and complex and therefore outside the scope of this discussion.

### Key knowledge gaps in PES studies

There has been a concerted research effort focused on forest owners’ preferences and their attitudes toward conservation. However, attitudes alone do not always translate to positive behavior or action. People will often have positive attitudes toward nature conservation, but when it comes to acting on these values, such as signing up for a conservation covenant, they stumble for variety of reasons; this effect is termed the *value–behavior gap* (Conte and Griffin 2019, Robb et al. 2019). Research on understanding the reasons landowners stumble on signing PES contracts for conservation is a key knowledge gap.

We discuss the potential immediate biophysical effects of climate-induced forest change on the METSO program. However, the effects of climate change extend further than ecological systems alone. A knowledge gap that needs to be urgently addressed for any future PES scheme design and or reforms is research on how exactly landowners understand the effects of climate change on their harvest or crop and whether the understanding translates to a shift toward or away from PES schemes. This will help anticipate future needs for setting targets and other design features that we discuss in this article.

Climate change may also bring other indirect effects on ecosystems that could affect PES. For example, in the boreal region there are projected biotic (e.g., insect outbreak) and abiotic (e.g., windstorm) factors that may affect forest ecosystems (Peltola et al. 2010, Venäläinen et al. 2020). There is evidence that forest owners are starting to shift their forest management practices in light of climate change (Blennow 2012, Vehola et al. 2022) already. This may change their attitude toward conservation contracts, especially if they see them as risks to future harvest yield. How the awareness of forest owners about the impacts of climate change on their harvest or production may change their attitude to voluntary protection contracts is an important research question that needs to be investigated urgently in Finland and globally. We found no research that directly investigates this chain of questions in the PES literature.

A consideration that we did not account for in our estimates of cost of protection of other effective conservation measures to reach EU biodiversity targets, is the feedback effect of voluntary forest conservation contracts on the supply of timber products (known as market equilibrium see Adamowicz et al. 2019). In addition, there will be subsequent effects on global market prices, which could then affect future conservation contracts; if other effective conservation measures such as METSO were to be used to reach EU biodiversity targets. We note a lack of discussion on this issue in the PES literature, although we acknowledge for such an effect to be a real concern the said program or policy will have to be many magnitudes larger than METSO and concern the whole of boreal Europe.

### Recommendations: METSO program and beyond

Enough time has passed to allow for pilot PES projects to finish, initial insights to be shared, and the PES literature to proliferate but apparently not long enough for a systematic reporting framework to be developed and adopted. This makes it difficult to synthesize and communicate research results. There are enough publications in this space to justify a conventional reporting requirement when assessing and communicating the effectiveness of PES schemes.

We demonstrate that compensation based on timber volume alone is an unreliable proxy for biodiversity value and therefore reduces the biodiversity return on investment and exposes the program to climate change related impacts that would result in higher compensation costs in the future. Our recommendation is to combine the information from initial site visits for assessing forest quality and use this as a biodiversity value proxy in combination with timber volume, so that there is a direct biodiversity-related measurement associated with the compensation. This also allows landowners who retain forest features such as deadwood and habitat trees to benefit from higher payments and their motivations to be rewarded.

As our case study from Finland demonstrates, supply and demand for PES schemes are not distributed evenly in space, particularly when climate change effects on land use and natural products such as timber become more pronounced. This requires more agile policy instruments such as introducing competitive contract mechanisms, especially when demand exceeds the supply of PES contracts. An example of such a policy instrument is reverse tendering, where landowners can place bids at an auction where they compete for the same contract for more land or conservation management actions (Naidoo and Adamowitz 2005, Murdoch et al. 2007, Ens 2012). Auction-based PES also reduces the information rent, the true opportunity cost to the landowner to take actions that benefit biodiversity or improve ecosystem services provision (Conte and Griffin 2019 and Banerjee and Conte 2018).

Offering certainty and permanence in contracts offered to landowners is vital to the success of PES schemes, as was discussed in the “Permanence” section. Deciding the future of one's forest or land parcel can often be a complex and time-consuming process. Under METSO, the option for fixed-term temporary contracts was intended to give landowners the time and flexibility they required before making final decisions about their forests (Terhi Koskela, Natural Resources Institute Finland, personal communication, 1 November 2023.). In small reserves, the ecological benefits of fixed-term temporary contracts over a 20–30-year period match to those of permanent contracts (Mönkkönen et al. 2011).

## Supplementary Material

biaf155_Supplemental_File

## Data Availability

All publicly available data used in this article are listed in table 2 (Appendix) with their associated links.

## Author Biography

Khorloo Batpurev (khorloo.batpurev@helsinki.fi) and Heini Kujala are affiliated with the Conservation Biology Informatics Group, Finnish Natural History Museum, in Helsinki, Finland. Khorloo Batpurev and Steve J. Sinclair are affiliated with the Department of Energy, Environment, and Climate Action, at the Arthur Rylah Institute for Environmental Research, in Heidelberg, Victoria, Australia. Khorloo Batpurev and Mar Cabeza are affiliated with the Global Change Conservation Lab, in the Faculty of Biological and Environmental Sciences at the University of Helsinki, in Helsinki, Finland. Kimmo Syrjänen is affiliated with the Finnish Environment Institute, in Helsinki, Finland. María Triviño is affiliated with the Department of Biological and Environmental Science and the School of Resource Wisdom at the University of Jyväskylä, in Jyväskylä, Finland.

## References

[bib1] Abildtrup J, Stenger A, de Morogues F, Polomé P, Blondet M, Michel C. 2021. Biodiversity protection in private forests: PES schemes, institutions and prosocial behavior. Forests 12: 1241.

[bib1a] Adamowicz W et al. 2019. Assessing ecological infrastructure investments. Proceedings of the National Academy of Sciences 116: 5254–5261. 10.1073/pnas.1802883116 PMC6431190 30617080

[bib2] Alrahahleh L, Ikonen VP, Kilpeläinen A, Torssonen P, Strandman H, Asikainen A, Kaurola J, Venäläinen A, Peltola H. 2016. Effects of forest conservation and management on volume growth, harvested amount of timber, carbon stock, and amount of deadwood in Finnish boreal forests under changing climate. Canadian Journal of Forest Research 47: 215–225. 10.1139/cjfr-2016-0153

[bib3] Armstrong C . 2019. Sharing conservation burdens fairly. Conservation Biology 33: 554–560. 10.1111/cobi.13260 30569477

[bib4] Balotari-Chiebao F, Santangeli A, Piirainen S, Byholm P. 2023. Wind energy expansion and birds: Identifying priority areas for impact avoidance at a national level. Biological Conservation 277: 109851. 10.1016/j.biocon.2022.109851

[bib2a] Banerjee S, Conte MN. 2018. Information access, conservation practice choice, and rent seeking in conservation procurement auctions: Evidence from a laboratory experiment. American Journal of Agricultural Economics 100: 1407–1426. 10.1093/ajae/aay064

[bib5] Blanco E, Moros L, Pfaff A, Steimanis I, Velez MA, Vollan B. 2023. No crowding out among those terminated from an ongoing PES program in Colombia. Journal of Environmental Economics and Management 120: 102826. 10.1016/j.jeem.2023.102826

[bib6] Blennow K . 2012. Adaptation of forest management to climate change among private individual forest owners in Sweden. Forest Policy and Economics 24: 41–47. 10.1016/j.forpol.2011.04.005

[bib7] Blicharska M, Orlikowska EH, Roberge J-M, Grodzinska-Jurczak M. 2016. Contribution of social science to large scale biodiversity conservation: A review of research about the Natura 2000 network. Biological Conservation 199: 110–122. 10.1016/j.biocon.2016.05.007

[bib8] Brownson K, Anderson EP, Ferreira S, Wenger S, Fowler L, German L. 2020. Governance of payments for ecosystem ecosystem services influences social and environmental outcomes in Costa Rica. Ecological Economics 174: 106659. 10.1016/j.ecolecon.2020.106659

[bib9] [CBD] Convention on Biological Diversity . 2014. Resourcing the Aichi Biodiversity Targets: An Assessment of Benefits, Investments, and Resource Needs for Implementing the Strategic Plan for Biodiversity 2011–2020. CBD.

[bib10] [CBD] Convention on Biological Diversity . 2022. The Kunming–Montreal Global Biodiversity Framework. CBD.

[bib3a] Conte MN, Griffin R. 2019. Private benefits of conservation and procurement auction performance. Environmental and Resource Economics 73: 759–790. 10.1007/s10640-019-00333-y.

[bib13] Daigneault A, Baker JS, Guo J, Lauri P, Favero A, Forsell N, Johnston C, Ohrel SB, Sohngen B. 2022. How the future of the global forest sink depends on timber demand, forest management, and carbon policies. Global Environmental Change 76: 102582. 10.1016/j.gloenvcha.2022.102582 PMC10631560 38024226

[bib70] Dzian M, Palus H, Parobek J, Lazarevic A. 2022. What are the implications of Ukraine conflict on the EU forest products trade? Paper presented at the 15th International Scientific Conference WoodEMA; 8–10 June 2022, Trnava , Slovak Republic.

[bib17] Engel S, Pagiola S, Wunder S. 2008. Designing payments for environmental services in theory and practice: An overview of the issues. Ecological Economics 65: 663–674. 10.1016/j.ecolecon.2008.03.011

[bib18] Ens EJ . 2012. Monitoring outcomes of environmental service provision in low socio-economic indigenous Australia using innovative CyberTracker technology. Conservation and Society 10: 42–52. www.jstor.org/stable/26393062

[bib19] Erbaugh JT . 2022. Impermanence and failure: The legacy of conservation-based payments in Sumatra, Indonesia. Environmental Research Letters 17: 054015. 10.1088/1748-9326/ac6437

[bib4a] ESRI . 2020. ArcGIS Desktop: Release 10.8.1. Environmental Systems Research Institute, Redlands.

[bib20] European Commission. 2020. EU Biodiversity Strategy for 2030: Bringing Nature Back into Our Lives. European Union. https://eur-lex.europa.eu/legal-content/EN/ALL/?uri=celex:52020DC0380

[bib12] European Committee of the Regions . 2020. Bio-diverse Cities and Regions beyond 2020 at the UN CBD COP 15 and in the EU Biodiversity Strategy for 2030. European Committee of the Regions, Commission for the Environment, Climate change and Energy.

[bib23] Ferraro PJ . 2017. Are payments for ecosystem services benefiting ecosystems and people? Pages 159–166 in Kareiva P et al., eds. Effective Conservation Science: Data Not Dogma. Oxford University Press. 10.1093/oso/9780198808978.003.0025

[bib24] Ford R, Williams K, Smith E, Bishop I. 2014. Beauty, belief, and trust: Toward a model of psychological processes in public acceptance of forest management. Environment and Behavior 46: 476–506. 10.1177/0013916512456023

[bib27] Forsius M et al. 2023. Modelling the regional potential for reaching carbon neutrality in Finland: Sustainable forestry, energy use and biodiversity protection. Ambio 52: 1757–1776. 10.1007/s13280-023-01860-1 37561360 PMC10562359

[bib28] Gómez-Baggethun E, de Groot R, Lomas PL, Montes C. 2010. The history of ecosystem services in economic theory and practice: From early notions to markets and payment schemes. Ecological Economics 69: 1209–1218. 10.1016/j.ecolecon.2009.11.007

[bib29] Gulbrandsen LH . 2014. Dynamic governance interactions: Evolutionary effects of state responses to non-state certification programs. Regulation and Governance 8: 74–92. 10.1111/rego.12005

[bib30] Hänninen H, Hamunen K, Viitala E-J, Kurttila M. 2021. Metsätalouden määräaikainen ympäristötuki: Mitä tapahtuu sopimusten päätyttyä? Metsätieteen Aikakauskirja Vuosikerta 2021: 10578. 10.14214/ma.10578

[bib31] Hanski I . 2000. Extinction debt and species credit in boreal forests: Modelling the consequences of different approaches to biodiversity conservation. Annales Zoologici Fennici 37: 271–280. http://www.jstor.org/stable/23735720

[bib33] Hiedanpää J . 2002. European-wide conservation versus local well-being: The reception of the Natura 2000 Reserve Network in Karvia, SW-Finland. Landscape and Urban Planning 61: 113–123. 10.1016/S0169-2046(02)00106-8

[bib34] Horne P . 2006. Forest owners’ acceptance of incentive based policy instruments in forest biodiversity conservation: A choice experiment based approach. Silva Fennica 40: 359.

[bib35] [IPBES] Intergovernmental Science-Policy Platform on Biodiversity and Ecosystem Service . 2019. Global Assessment Report of the Intergovernmental Science-Policy Platform on Biodiversity and Ecosystem Service. IPBES.

[bib5a] Jayachandran S, de Laat J, Lambin EF, Stanton CY, Audy R, Thomas NE. 2017. Cash for carbon: A randomized trial of payments for ecosystem services to reduce deforestation. Science 357: 267–273. 10.1126/science.aan0568 28729505

[bib37] Kallio AMI, Hänninen R, Vainikainen N, Luque S. 2008. Biodiversity value and the optimal location of forest conservation sites in Southern Finland. Ecological Economics 67: 232–243. 10.1016/j.ecolecon.2008.05.005

[bib38] Kangas J, Ollikainen M. 2022. A PES scheme promoting forest biodiversity and carbon sequestration. Forest Policy and Economics 136: 102692. 10.1016/j.forpol.2022.102692

[bib41] Kati V, Hovardas T, Dieterich M, Ibisch PL, Mihok B, Selva N. 2015. The challenge of implementing the European network of protected areas Natura 2000. Conservation Biology 29: 260–270. 10.1111/cobi.12366 25103194

[bib42] Knight AT, Grantham HS, Smith RJ, McGregor GK, Possingham HP, Cowling RM. 2011. Land managers’ willingness-to-sell defines conservation opportunity for protected area expansion. Biological Conservation 144: 2623–2630. 10.1016/j.biocon.2011.07.013

[bib44] Korhonen KT et al. 2021. Forests of Finland 2014–2018and their development 1921–2018. Silva Fennica 55: 10662. 10.14214/sf.10662

[bib46] Koskela T, Karppinen H. 2021. Forest owners’ willingness to implement measures to safeguard biodiversity: Values, attitudes, ecological worldview, and forest ownership objectives. Small-Scale Forestry 20: 11–37. 10.1007/s11842-020-09454-5

[bib45] Koskela T, Anttila S, Aapala K, Muttilainen H. 2024. METSO Status Report 2023: Southern Finland Forest Biodiversity Action Plan 2008–2025. Finland's Environmental Institute.

[bib48] Kotiaho JSA et al. 2021. Metsäluonnon Turvaava Suojelun Kohdentaminen Suomessa. Suomen Luontopaneeli.

[bib49] Kouki J, Löfman S, Martikainen P, Rouvinen S, Uotila A. 2001. Forest fragmentation in Fennoscandia: Linking habitat requirements of wood-associated threatened species to landscape and habitat changes. Scandinavian Journal of Forest Research 16: 27–37. 10.1080/028275801300090564

[bib50] Kroeger T . 2013. The quest for the “optimal” payment for environmental services program: Ambition meets reality, with useful lessons. Forest Policy and Economics 37: 65–74. 10.1016/j.forpol.2012.06.007

[bib52] Kulju I, Niinistö T, Peltola A, Räty M, Sauvula-Seppälä T, Torvelainen J, Uotila E, Vaahtera E. 2022. Finnish Statistical Yearbook of Forestry. Finnish Natural Resources Institute.

[bib54] Kuusela S et al., eds. 2022. Kohti Kattavaa Suojelualueverkostoa: Luonnon Monimuotoisuuden Turvaamisen Painopisteet Suomessa. Finnish Environment Institute. Suomen ympäristökeskuksen raportteja no. 18/2022 https://helda.helsinki.fi/items/f2e6cbb6-a773-464b-abed-b23f3b4f03c3

[bib55] Lambin EF et al. 2014. Effectiveness and synergies of policy instruments for land use governance in tropical regions. Global Environmental Change 28: 129–140. 10.1016/j.gloenvcha.2014.06.007

[bib56] Lassauce A, Paillet Y, Jactel H, Bouget C. 2011. Deadwood as a surrogate for forest biodiversity: Meta-analysis of correlations between deadwood volume and species richness of saproxylic organisms. Ecological Indicators 11: 1027–1039. 10.1016/j.ecolind.2011.02.004

[bib59] Lister J . 2011. Corporate Social Responsibility and the State: International Approaches to Forest Co-Regulation. UBC Press. https://books.google.com.au/books?id=2M5TCgAAQBAJ

[bib60] Lliso B, Arias-Arévalo P, Maca-Millán S, Engel S, Pascual U. 2022. Motivational crowding effects in payments for ecosystem services: Exploring the role of instrumental and relational values. People and Nature 4: 312–329. 10.1002/pan3.10280

[bib61] Löfroth T, Birkemoe T, Shorohova E, Dynesius M, Fenton NJ, Drapeau P, Tremblay JA. 2023. Deadwood biodiversity. Pages 167–189 in Girona MM, Morin H, Gauthier S, Bergeron Y eds. Boreal Forests in the Face of Climate Change: Sustainable Management. Springer. 10.1007/978-3-031-15988-6_6

[bib62] Luck GW, Chan KMA, Eser U, Gómez-Baggethun E, Matzdorf B, Norton B, Potschin MB. 2012. Ethical considerations in on-ground applications of the ecosystem services concept. BioScience 62: 1020–1029. 10.1525/bio.2012.62.12.4

[bib63] Mahanty S, Suich H, Tacconi L. 2013. Access and benefits in payments for environmental services and implications for REDD+: Lessons from seven PES schemes. Land Use Policy 31: 38–47. 10.1016/j.landusepol.2011.10.009

[bib66] Matthews N, Missingham B. 2009. Social accountability and community forest management: The failure of collaborative governance in the Wombat Forest. Development in Practice 19: 1052–1063. 10.1080/09614520903220800

[bib69] McDonald JA, Helmstedt KJ, Bode M, Coutts S, McDonald-Madden E, Possingham HP. 2018. Improving private land conservation with outcome-based biodiversity payments. Journal of Applied Ecology 55: 1476–1485. 10.1111/1365-2664.13071

[bib72] Mikkonen N, Leikola N, Lehtomäki J, Halme P, Moilanen A. 2023. National high-resolution conservation prioritisation of boreal forests. Forest Ecology and Management 541: 121079. 10.1016/j.foreco.2023.121079

[bib73] Miljand M, Bjärstig T, Eckerberg K, Primmer E, Sandström C. 2021. Voluntary agreements to protect private forests: A realist review. Forest Policy and Economics 128: 102457. 10.1016/j.forpol.2021.102457

[bib75] Mönkkönen M, Reunanen P, Kotiaho JS, Juutinen A, Tikkanen O-P, Kouki J. 2011. Cost-effective strategies to conserve boreal forest biodiversity and long-term landscape-level maintenance of habitats. European Journal of Forest Research 130: 717–727. 10.1007/s10342-010-0461-5

[bib76] Morita K, Matsumoto K. 2023. Challenges and lessons learned for REDD+ finance and its governance. Carbon Balance and Management 18: 8. 10.1186/s13021-023-00228-y 37199889 PMC10193719

[bib77] Murdoch W, Polasky S, Wilson KA, Possingham HP, Kareiva P, Shaw R. 2007. Maximizing return on investment in conservation. Biological Conservation 139: 375–388. 10.1016/j.biocon.2007.07.011

[bib79] Naidoo R, Adamowicz WL. 2005. Economic benefits of biodiversity exceed costs of conservation at an African rainforest reserve. Proceedings of the National Academy of Sciences 102: 16712–16716. 10.1073/pnas.0508036102 PMC1283836 16267131

[bib82] Pagiola S . 2011. Using PES to Implement REDD. Latin America and Caribbean Sustainable Development Department, World Bank. PES learning paper no. 2011-1.

[bib83] Paletto A, Báliková K, De Meo I. 2021. Opinions towards the water-related payments for ecosystem services (PES) schemes: The stakeholders’ point of view. Water and Environment Journal 35: 1051–1062. 10.1111/wej.12697

[bib84] Parisi F, Pioli S, Lombardi F, Fravolini G, Marchetti M, Tognetti R. 2018. Linking deadwood traits with saproxylic invertebrates and fungi in European forests: A review iForest: Biogeosciences and Forestry 11: 423–436. 10.3832ifor2670-011

[bib85] Peltola H, Ikonen VP, Gregow H, Strandman H, Kilpeläinen A, Venäläinen A, Kellomäki S. 2010. Impacts of climate change on timber production and regional risks of wind-induced damage to forests in Finland. Forest Ecology and Management 260: 833–845. 10.1016/j.foreco.2010.06.001

[bib6a] Persson MU, Alpizar Rodriguez F. 2013. Conditional cash transfers and payments for environmental services—A conceptual framework for explaining and judging differences in outcomes. World Development 43: 124–137. https://EconPapers.repec.org/RePEc:eee:wdevel:v:43:y:2013:i:c:p:124-137

[bib88] Primmer E, Paloniemi R, Similä J, Tainio A. 2014. Forest owner perceptions of institutions and voluntary contracting for biodiversity conservation: Not crowding out but staying out. Ecological Economics 103: 1–10. 10.1016/j.ecolecon.2014.04.008

[bib89] Pukkala T, Vauhkonen J, Korhonen KT, Packalen T. 2021. Self-learning growth simulator for modelling forest stand dynamics in changing conditions. Forestry 94: 333–346. 10.1093/forestry/cpab008

[bib90] Ranius T, Korosuo A, Roberge J-M, Juutinen A, Mönkkönen M, Schroeder M. 2016. Cost-efficient strategies to preserve dead wood-dependent species in a managed forest landscape. Biological Conservation 204: 197–204. 10.1016/j.biocon.2016.10.017

[bib91] Reeson AF, Rodriguez LC, Whitten SM, Williams K, Nolles K, Windle J, Rolfe J. 2011. Adapting auctions for the provision of ecosystem services at the landscape scale. Ecological Economics 70: 1621–1627. 10.1016/j.ecolecon.2011.03.022

[bib92] Robb J, Haggar J, Lamboll R, Castellanos E. 2019. Exploring the value–action gap through shared values, capabilities and deforestation behaviours in Guatemala. Environmental Conservation 46: 226–233. 10.1017/S0376892919000067

[bib93] Rode J, Gómez-Baggethun E, Krause T. 2015. Motivation crowding by economic incentives in conservation policy: A review of the empirical evidence. Ecological Economics 117: 270–282. 10.1016/j.ecolecon.2014.11.019

[bib94] Rosa da Conceição H, Börner J, Wunder S. 2015. Why were upscaled incentive programs for forest conservation adopted? Comparing policy choices in Brazil, Ecuador, and Peru. Ecosystem Services 16: 243–252. 10.1016/j.ecoser.2015.10.004

[bib95] Salzman J, Bennett G, Carroll N, Goldstein A, Jenkins M. 2018. The global status and trends of payments for ecosystem services. Nature Sustainability 1: 136–144. 10.1038/s41893-018-0033-0

[bib96] Sierra R, Russman E. 2006. On the efficiency of environmental service payments: A forest conservation assessment in the Osa Peninsula, Costa Rica. Ecological Economics 59: 131–141. 10.1016/j.ecolecon.2005.10.010

[bib97] Sinclair SJ, Avirmed O, White MD, Batpurev K, Griffioen PA, Liu C, Jambal S, Sime H, Olson KA. 2021. Rangeland condition assessment in the Gobi Desert: A quantitative approach that places stakeholder evaluations front and centre. Ecological Economics 181: 106891. 10.1016/j.ecolecon.2020.106891

[bib98] Sironen S, Primmer E, Leskinen P, Similä J, Punttila P. 2020. Context sensitive policy instruments: A multi-criteria decision analysis for safeguarding forest habitats in Southwestern Finland. Land Use Policy 92: 104460. 10.1016/j.landusepol.2019.104460

[bib100] Song C, Liu Y, Liu L, Xian C, Wang X. 2023. A scientometric analysis of payments for ecosystem services research: Mapping global trends and directions. Sustainability 15: 15649.

[bib101] Stokland J, Tomter SM, Söderberg U. 2004. Development of dead wood indicators for biodiversity monitoring: Experiences from Scandinavia. EFI Proceedings 51: 207–226.

[bib102] Stokland JN, Siitonen J, Jonsson BG. 2012. Biodiversity in dead wood. Cambridge University Press. 10.1017/CBO9781139025843

[bib105] Thompson BS . 2021. Corporate payments for ecosystem services in theory and practice: Links to economics, business, and sustainability. Sustainability 13: 8307.

[bib106] Tiebel M, Mölder A, Plieninger T. 2021. Small-scale private forest owners and the European Natura 2000 conservation network: Perceived ecosystem services, management practices, and nature conservation attitudes. European Journal of Forest Research 140: 1515–1531. 10.1007/s10342-021-01415-7

[bib107] Triviño M, Morán-Ordoñez A, Eyvindson K, Blattert C, Burgas D, Repo A, Pohjanmies T, Brotons L, Snäll T, Mönkkönen M. 2023a. Future supply of boreal forest ecosystem services is driven by management rather than by climate change. Global Change Biology 29: 1484–1500. 10.1111/gcb.16566 36534408

[bib108] Triviño M, Potterf M, Tijerín J, Ruiz-Benito P, Burgas D, Eyvindson K, Blattert C, Mönkkönen M, Duflot R. 2023b. Enhancing resilience of boreal forests through management under global change: A review. Current Landscape Ecology Reports 8: 103–118. 10.1007/s40823-023-00088-9

[bib7a] UNEP-WCMC and IUCN . 2021. Protected Planet: the World Database on Protected Areas (WDPA)/Database on Other Effective Area-based Conservation Measures. [September/2021], Cambridge, UK: UNEP-WCMC and IUCN. Available at: www.protectedplanet.net.

[bib110] Vehola A, Malkamäki A, Kosenius A-K, Hurmekoski E, Toppinen A. 2022. Risk perception and political leaning explain the preferences of non-industrial private landowners for alternative climate change mitigation strategies in Finnish forests. Environmental Science and Policy 137: 228–238. 10.1016/j.envsci.2022.09.003

[bib111] Venäläinen A, Lehtonen I, Laapas M, Ruosteenoja K, Tikkanen O-P, Viiri H, Ikonen V-P, Peltola H. 2020. Climate change induces multiple risks to boreal forests and forestry in Finland: A literature review. Global Change Biology 26: 4178–4196. 10.1111/gcb.15183 32449267 PMC7383623

[bib112] Virkkala R, Leikola N, Kujala H, Kivinen S, Hurskainen P, Kuusela S, Valkama J, Heikkinen RK. 2022. Developing fine-grained nationwide predictions of valuable forests using biodiversity indicator bird species. Ecological Applications 32: e2505. 10.1002/eap.2505 34866270 PMC9285730

[bib113] Vorlaufer T, de Laat J, Engel S. 2023. Do payments for environmental services affect forest access and social preferences in the long run? Experimental evidence from Uganda. Journal of the Association of Environmental and Resource Economists 10: 389–412. 10.1086/721440

[bib115] Westholm L, Ostwald M, Henders S, Mattsson E. 2011. Learning from Norway: A Review of Lessons Learned for REDD+ Donors. Forest, Climate, and Livelihood Research Network.

[bib116] Winkel G et al. 2022. Governing Europe's forests for multiple ecosystem services: Opportunities, challenges, and policy options. Forest Policy and Economics 145: 102849. 10.1016/j.forpol.2022.102849

[bib117] Wuepper D, Huber R. 2022. Comparing effectiveness and return on investment of action- and results-based agri-environmental payments in Switzerland. American Journal of Agricultural Economics 104: 1585–1604. 10.1111/ajae.12284

[bib119] Wunder S, Engel S, Pagiola S. 2008. Taking stock: A comparative analysis of payments for environmental services programs in developed and developing countries. Ecological Economics 65 834–852. 10.1016/j.ecolecon.2008.03.010

[bib118] Wunder S, Börner J, Ezzine-de-Blas D, Feder S, Pagiola S. 2020. Payments for environmental services: Past performance and pending potentials. Annual Review of Resource Economics 12: 209–234. 10.1146/annurev-resource-100518-094206

